# Oxidative Stress Attenuates TLR3 Responsiveness and Impairs Anti-viral Mechanisms in Bronchial Epithelial Cells From COPD and Asthma Patients

**DOI:** 10.3389/fimmu.2019.02765

**Published:** 2019-11-29

**Authors:** Mandy Menzel, Sangeetha Ramu, Jenny Calvén, Beata Olejnicka, Asger Sverrild, Celeste Porsbjerg, Ellen Tufvesson, Leif Bjermer, Hamid Akbarshahi, Lena Uller

**Affiliations:** ^1^Unit of Respiratory Immunopharmacology, Department of Experimental Medical Science, Lund University, Lund, Sweden; ^2^Department of Internal Medicine, University of Gothenburg, Gothenburg, Sweden; ^3^Airway Inflammation Unit, Department of Experimental Medical Science, Lund University, Lund, Sweden; ^4^Department of Internal Medicine, Trelleborg Hospital, Trelleborg, Sweden; ^5^Department of Respiratory Medicine, Bispebjerg and Frederiksberg Hospital, Copenhagen, Denmark; ^6^Unit of Respiratory Medicine and Allergology, Department of Clinical Sciences Lund, Lund University, Lund, Sweden

**Keywords:** asthma, COPD, bronchial epithelium, oxidative stress, rhinovirus, interferon, pattern recognition receptors

## Abstract

COPD and asthma exacerbations are commonly triggered by rhinovirus infection. Potentially promoting exacerbations, impaired anti-viral signaling and attenuated viral clearance have been observed in diseased bronchial epithelium. Oxidative stress is a feature of inflammation in asthma and COPD and is prominent during exacerbations. It is not known whether oxidative stress affects the anti-viral signaling capacity. Bronchial epithelial cells from asthmatic and COPD donors were infected with rhinovirus or treated with the oxidative stressor H_2_O_2_ followed by exposure to the synthetic viral replication intermediate poly(I:C). Poly(I:C) was used to ascertain a constant infection-like burden. Gene and protein levels of antioxidants as well as anti-viral responses were measured 3 and 24 h post poly(I:C) exposure. Rhinovirus infection and poly(I:C) stimulation induced protein levels of the antioxidants SOD1 and SOD2. In asthmatic bronchial epithelial cells pre-treatment with H_2_O_2_ dose-dependently decreased the antioxidant response to poly(I:C), suggesting exaggerated oxidative stress. Further, poly(I:C)-induced IFNβ gene expression was reduced after pre-treatment with H_2_O_2_. This epithelial effect was associated with a reduced expression of the pattern recognition receptors RIG-I, MDA5 and TLR3 both on gene and protein level. Pre-treatment with H_2_O_2_ did not alter antioxidant responses in COPD bronchial epithelial cells and, more modestly than in asthma, reduced poly(I:C)-induced IFNβ gene expression. Knockdown of TLR3 but not RIG-I/MDA5 abrogated impairment of poly(I:C)-induced IFNβ gene expression by H_2_O_2_. We developed a method by which we could demonstrate that oxidative stress impairs anti-viral signaling in bronchial epithelial cells from asthmatic and COPD patients, most pronounced in asthma. The impairment apparently reflects reduced responsiveness of TLR3. These present findings shed light on molecular mechanisms potentially causing reduced interferon responses to rhinovirus infection at exacerbations in asthma and COPD. Together, our findings suggest a possible self-perpetuating vicious cycle underlying recurrent exacerbations, leading to an impaired anti-viral response, which in turn leads to viral-induced exacerbations, causing more airway inflammation.

## Introduction

Asthma and COPD are chronic diseases of the airways, characterized by inflammation of the bronchi and airflow obstruction. Disease severity may be acutely increased at periods defined as exacerbations, which also may be involved in disease progression (1, 2). Exacerbations are a major cause of morbidity and mortality in asthma, but at present, it is unclear why some patients have frequent exacerbations, while others rarely, or never experience exacerbations (3). Understanding the mechanisms driving recurrent exacerbations is crucial to the development of better management strategies. Infections with rhinoviruses are a main cause of exacerbations of asthma and COPD. The airway epithelium is the main target of rhinoviruses. The epithelial cells express a variety of pattern recognition receptors (PRRs), such as TLR3 and the RIG-I like helicases RIG-I and MDA5. PRRs recognize viruses and mount immunological responses increasing epithelial production of inflammatory cytokines, chemokines, and anti-viral interferons (4, 5). Several studies have demonstrated a reduced anti-viral response toward rhinoviral infection, leading to a higher viral burden in asthmatics (6–8).

The airway inflammation at exacerbations of asthma and COPD likely involves oxidative stress (9, 10). Inflammatory cells participating in inflammatory processes in these diseases, such as alveolar macrophages, eosinophils and neutrophils, are known sources of reactive oxygen species and free radicals (11). Oxidative stress is caused by an imbalance between the oxidant and the antioxidant system, in which free radicals overcome the cell's antioxidant defenses (12). Oxidative stress not only acts as an inflammatory trigger, likely by activation of the transcription factor NFκB (13), but might also lead to smooth muscle hyper-contractility (14). Both environmental and genetic factors contribute to oxidative stress. Polymorphisms in the gene coding for glutathione-S-transferase, which is involved in maintaining physiological oxidant levels in the cell, have been associated with asthma (15). Further, it has been demonstrated that proteases from allergens can induce production of reactive oxygen species (16) and oxidative stress produced by cigarette smoke is considered a major disease mechanism in COPD (17).

Infection with rhinovirus causes oxidative stress (18), probably through interaction with a NOD-like receptor (19, 20). The dsRNA mimic poly(I:C) induces oxidative stress by the same mechanism (19, 20) supporting the view that dsRNA is responsible for reactive oxygen production at rhinoviral infections. A previous study suggested that increased mitochondrial oxygen species could decrease early TLR9 mediated type I interferons while increasing a second wave of type I interferons mediated via RIG-I signaling in plasmacytoid dendritic cells (21).

It is yet largely unknown how oxidative stress affects the anti-viral signaling cascade in the major bronchial target for infection, the epithelium. Here we demonstrate that the oxidative stressor H_2_O_2_ reduces dsRNA-induced expression of IFNβ and pattern recognition receptors in bronchial epithelial cells of asthmatics and to some extent also in COPD patients. Further, we provide evidence that the effect of H_2_O_2_ on anti-viral responses might involve TLR3.

## Materials and Methods

### Primary Bronchial Epithelial Cells

Primary human bronchial epithelial cells (HBECs) from asthmatic and COPD donors were obtained by bronchoscopy using a fiber optic bronchoscope with standard sterile-sheared nylon cytology brushes. Brushings were performed in accordance with standard guidelines and processed as described previously (22). Patients provided written informed consent and the regional ethical review board in Lund and the review board in Copenhagen approved the study. A characterization of patients can be found in Tables 1, 2.

**Table 1:** Patient characteristics of asthmatics used for the oxidative stress model.

**FEV_**1**_ [%pred]**	**Atopy**	**Concomitant medication**
94.0	No	LTRA, LABA
67.7	Yes	ICS, LABA, SABA
107.3	Yes	ICS, LABA
54.0	Yes	ICS, LABA
99.0	Yes	ICS, LABA
88.0	Yes	ICS, LABA, LTRA
86.0	Yes	ICS, LABA, LTRA
80.0	Yes	ICS, LABA, LAMA

*Spirometry data was obtained without administration of bronchodilators*.

*Samples were obtained from 5 female and 3 male patients with an age span of 21–60 years (median 46 years)*.

*FEV_1_, forced expiratory volume in 1s; ICS, inhaled corticosteroids; LTRA, leukotriene receptor antagonists; LABA, long-acting beta-adrenoceptor agonist; SABA, short-acting beta-adrenoceptor agonist; LAMA, long-acting muscarinic antagonist*.

**Table 2:** Patient characteristics of COPD donors used for the oxidative stress model.

**FEV_**1**_ [%pred]**	**Pack years**	**Concomitant medication**
20.0^*^	40	NA
24.3^*^	50	NA
73.0	14	SABA, LAMA
74.0	47	none
44.0	45	LABA
62.0	42	LAMA

*Spirometry data was obtained without administration of bronchodilators*.

*Samples were obtained from 1 female and 5 male patients with an age span of 57–66 years (median 63 years)*.

*FEV_1_, forced expiratory volume in 1s; LABA, long-acting beta-adrenoceptor agonist; LAMA, long-acting muscarinic antagonist; SABA, short-acting beta-adrenoceptor agonist*.

* *For these donors bronchial epithelial cells were obtained by protein digestion of explanted lungs. For details see Calven et al. (23)*.

### Rhinovirus Infection

Rhinovirus RV16 was amplified in Ohio HeLa cells (European Collection of Cell Cultures) as described previously (24) and obtained from clarified cell lysates. HBECs were infected with RV16 at 1MOI (TCID_50_ 1.58 × 10^5^) for 1 h at room temperature while shaking. Then virus was removed and fresh culture medium was added. After 48 h cell lysates were collected for subsequent protein expression analysis. A characterization of patients can be found in Table 3.

**Table 3:** Patient characteristics of asthmatics used for measuring antioxidant levels after rhinovirus infection.

**FEV_**1**_ [%pred]**	**Atopy**	**Concomitant medication**
119.8	Yes	SABA
111.7	Yes	ICS, SABA
103.9	Yes	SABA
103.4	Yes	SABA
91.0	Yes	SABA
82.1	Yes	SABA
94.8	Yes	SABA

*Spirometry data was obtained without administration of bronchodilators*.

*Samples were obtained from 7 female patients with an age span of 20–32 years (median 25 years)*.

*FEV_1_, forced expiratory volume in 1s; ICS, inhaled corticosteroids; SABA, short-acting beta-adrenoceptor agonist*.

### Stimulation With H_2_O_2_ and Poly(I:C)

HBECs were cultured in bronchial epithelial growth medium (BEGM, Clonetics, San Diego, CA, USA or PneumaCult-Ex Medium, STEMCELL Technologies, Cambridge, UK) and subsequently seeded into 12-well plates (Nunc Technologies, Carlsbad, CA, USA). For experiments cells were exposed to H_2_O_2_ (Sigma-Aldrich, Stockholm, Sweden) for 30 min before stimulation with 10 μg/ml poly(I:C) (InvivoGen, San Diego, CA, USA) for 3 and 24 h. Cell lysates were obtained for gene and protein expression analysis and cell-free supernatants were obtained for analysis of protein release.

### Knockdown of Pattern Recognition Receptors

In subsequent experiments HBECs were transfected with 10 nM siRNA directed against MDA5, RIG-I or TLR3 or non-specific siRNA (scramble) using Lipofectamine RNAiMAX (Ambion, Thermo Scientific, Waltham, NA, USA) as a transfection agent prior to stimulation with H_2_O_2_ and poly(I:C).

### RNA Isolation and Quantification of Gene Expression

Total RNA was extracted using a RNA isolation kit (Nucleospin® RNA II, Macherey-Nagel, Düren, Germany) and 1 μg RNA was reverse transcribed to cDNA (Precision Nanoscript Reverse Transcription Kit, PrimerDesign, Southampton, UK). Quantitative real-time PCR was performed for real-time detection of PCR products on a Mx3005P qPCR system (Stratagene, La Jolla, CA, USA) using standard cycling parameters. Primers were obtained from Qiagen (Sollentuna, Sweden) or PrimerDesign (Southampton, UK). Target genes were normalized to UBC/GAPDH expression and related to unstimulated control using the ΔΔCt method (25).

### Quantification of Protein Expression by Western Blot

For western blot quantification cells were lysed in a lysis buffer containing 1% TritonX-100, 10 mM Tris-HCl, 50 mM NaCl, 5 mM EDTA, 30 mM Na_4_P_2_O_7_, 50 mM NaF, 0.1 mM Na_3_VO_4_, 1% phosphatase and protease inhibitors (Sigma-Aldrich, Stockholm, Sweden). Protein concentration were measured by BCA protein assay (Pierce, Thermo Scientific, Waltham, MA, USA) and equal amounts of protein were loaded and electrophoresed on a 4–20% TGX stain-free gel (Bio-Rad Laboratories AB, Solna, Sweden) for each sample. This was followed by blotting on a Trans-Blot Turbo Transfer System (Bio-Rad Laboratories AB, Solna, Sweden) and blocking of the membrane in 5% (w/v) milk in Tris-buffered saline Tween-20 and overnight incubation at 4°C with primary antibodies (anti-TLR3 Rabbit mAb, anti-RIG-I Rabbit mAb, anti-MDA5 Rabbit mAb, anti-SOD2 Rabbit pAb, anti-SOD1 Mouse mAb, anti-catalase Mouse mAb, anti-TRX Mouse mAb and anti-GAPDH Rabbit mAb; Cell Signaling Technology, Leiden, The Netherlands, Novocastra, Newcastle upon Tyne, UK, Sigma-Aldrich, Stockholm, Sweden and Mabtech AB, Nacka Strand, Sweden). Then the membrane was washed and incubated for 1 h with secondary antibodies (anti Rabbit IgG HRP-linked Ab and anti Mouse IgG HRP-linked Ab; Cell Signaling Technology, Leiden, The Netherlands). Chemiluminescent detection was performed using Clarity Max Western ECL Substrate (Bio-Rad Laboratories AB, Solna, Sweden) and immunoblots were visualized by LI-COR Odyssey Fc Imager (LI-COR Biosciences, Lincoln, NE, USA) and Image Studio (v3.1.4; LI-COR Biosciences, Lincoln, NE, USA).

### Protein Quantification by Multiplex ELISA

Levels of cytokines IFNβ, IL-1β, TNF, IL-8, IL-6, IL-33, and IL-17A were measured in cell-free supernatants by Luminex immunoassay according to the manufacturer's instructions (R&D Systems, Abingdon, UK). Data was acquired on a calibrated and validated Luminex MAGPIX instrument (R&D Systems, Abingdon, UK) as per manufacturer's instructions. The lower limit of quantification was 3.86 pg/ml for IFNβ, 3.39 pg/ml for IL-1β, 11.77 pg/ml for TNF, 2.10 pg/ml for IL-8, 9.19 pg/ml for IL-6, 15.25 pg/ml for IL-33, and 10.12 pg/ml for IL-17A.

### Cell Viability

Levels of lactate dehydrogenase were measured in cell-free supernatants according to the manufacturer's instructions (Roche Diagnostics, Bromma, Sweden) and related to total protein content in the supernatant (Pierce, Thermo Scientific, Waltham, MA, USA).

### Statistical Analysis

Data is presented as mean with standard error of the mean. Comparison between different groups was performed by Kruskal-Wallis with Wilcoxon post-testing. *P*-values < 0.05 were regarded statistically significant. Statistical analysis was performed using R (26).

## Results

### Rhinovirus Infection, as Well as Poly(I:C), Induces Superoxide Dismutase Protein Levels in Bronchial Epithelial Cells From Asthmatic Donors

**Figure 1:**
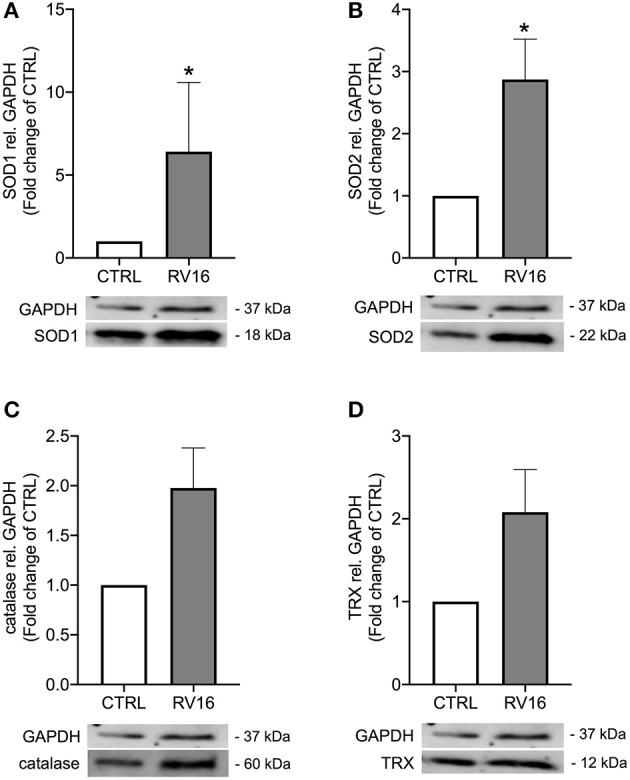
Rhinovirus infection induces expression of antioxidants in asthma bronchial epithelial cells. HBECs from asthma patients were infected with 1MOI RV16 for 48 h. Immunoblots of cell lysates were probed with anti-SOD1 **(A)**, anti-SOD2 **(B)**, anti-catalase **(C)**, and anti-TRX **(D)** and quantified. Data is presented as mean ± standard error of the mean (SEM) fold change of un-stimulated control relative to GAPDH expression. Comparison of different groups was performed by Kruskal-Wallis with Wilcoxon post-testing. *p < 0.05 vs. CTRL. Data was obtained from 7 donors.

**Figure 2:**
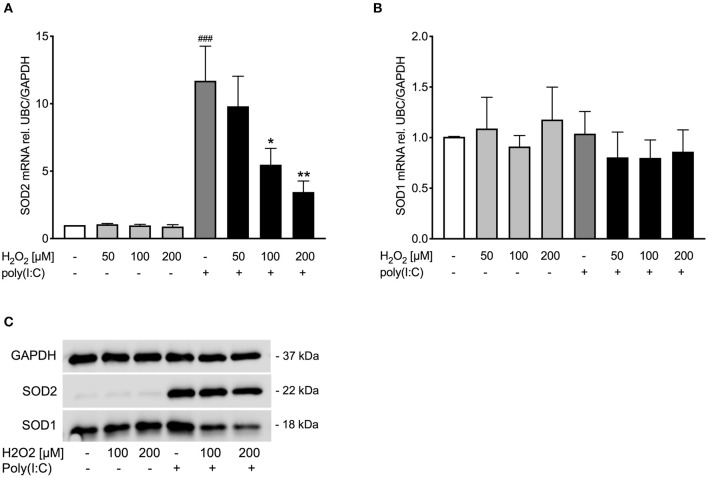
Poly(I:C)-induced SOD1 and SOD2 expression is reduced upon pre-treatment with H_2_O_2_ in asthma bronchial epithelial cells. HBECs from asthma patients were pre-treated with H_2_O_2_ for 30 min followed by stimulation with poly(I:C). Cells were harvested for gene and protein expression analysis after 3 and 24 h, respectively. Gene expression levels of SOD2 **(A)** and SOD1 **(B)** were measured by real-time PCR and data is presented as mean ± standard error of the mean (SEM) fold change of unstimulated control relative to UBC/GAPDH expression. Comparison of different groups was performed by Kruskal-Wallis with Wilcoxon post-testing. ^###^*p* < 0.001 vs. un-stimulated control (CTRL); *p < 0.05, **p < 0.01 vs. poly(I:C). Data was obtained from 7 donors. A representative western blot image (data from 2 donors) of SOD2 and SOD1 **(C)** protein is shown.

As rhinovirus infection has been shown to act as an oxidative stressor (18) and thus induces antioxidant expression, we investigated expression of four well-known antioxidants after rhinovirus infection in bronchial epithelial cells of asthmatics. Infection with rhinovirus significantly induced protein levels of manganese-dependent superoxide dismutase (SOD2) and copper-zinc superoxide dismutase (SOD1) (*p* < 0.05; Figures 1A,B). There was only a tendential up-regulation of catalase and thioredoxin (TRX) protein levels after rhinovirus infection (Figures 1C,D). Next asthma bronchial epithelial cells were stimulated with the synthetic rhinovirus replication intermediate poly(I:C). Similarly to rhinovirus infection, stimulation with poly(I:C) induced gene and protein expression of SOD1 and SOD2 (Figure 2).

### Poly(I:C)-Induced Superoxide Dismutase Expression Is Reduced in H_2_O_2_-Exposed Bronchial Epithelial Cells From Asthmatics

During exacerbations, frequently involving viral infections, an increase of levels of reactive oxygen species has been observed (27, 28). It was thus of interest to develop an *in vitro* model that could mimic such biphasic effects. Hence, bronchial epithelial cells from asthmatic donors were first given increasing doses of the oxidative stressor H_2_O_2_ which was then followed by stimulation with poly(I:C). Doses were chosen according to literature (29, 30). We regarded the present employment of poly(I:C) a suitable mode for explorative studies as biological actions of rhinovirus infection are mimicked (31). Further, by using poly(I:C) one can avoid variations due to take off rates of infection, thus, supporting reproducibility of data.

Pre-treatment with H_2_O_2_ dose-dependently reduced poly(I:C)-induced SOD2 expression both on gene (Figure 2A) and protein level (Figure 2C), while there was no effect of H_2_O_2_ on SOD1 gene expression (Figure 2B). Protein levels of poly(I:C)-induced SOD1 were reduced by H_2_O_2_ pre-treatment (Figure 2C). This data suggests that asthmatic epithelium subjected to oxidative stress may exhibit an imbalance between oxidant and antioxidant systems at viral infection.

### Pre-treatment With H_2_O_2_ Dose-Dependently Decreased Poly(I:C)-Induced IFNβ and TLR3 Expression in Asthma Bronchial Epithelial Cells

**Figure 3:**
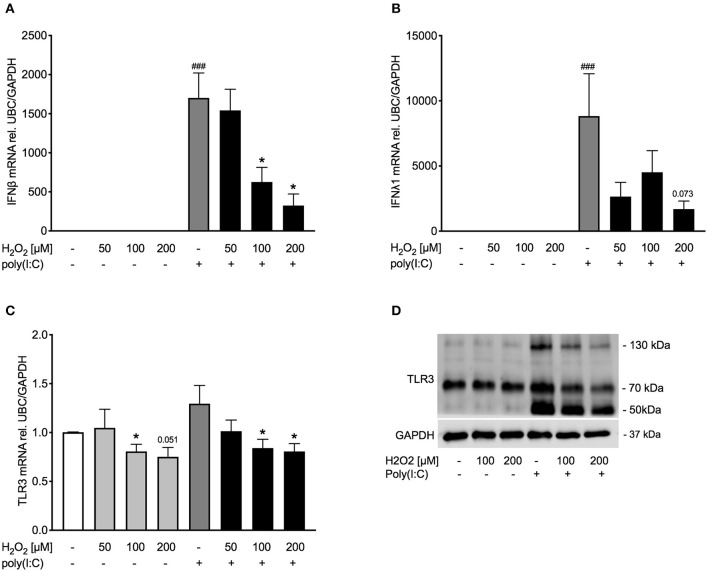
Pre-treatment with H_2_O_2_ reduces poly(I:C)-induced expression of IFNβ and IFNλ and decreases TLR3 expression. HBECs from asthma patients were pre-treated with H_2_O_2_ for 30 min followed by stimulation with poly(I:C). Cells were harvested for gene and protein expression analysis after 3 and 24 h, respectively. Gene expression levels of IFNβ **(A)**, IFNλ **(B)**, and TLR3 **(C)** were measured by real-time PCR and data is presented as mean ± standard error of the mean (SEM) fold change of unstimulated control relative to UBC/GAPDH expression. Comparison of different groups was performed by Kruskal-Wallis with Wilcoxon post-testing. ^###^*p* < 0.001 vs. un-stimulated (CTRL); *p < 0.05 vs. poly(I:C). Data was obtained from 7 donors. A representative western blot image (data from 2 donors) of TLR3 **(D)** protein is shown.

After establishing a model of oxidative stress, we intended to investigate whether oxidative stress affects anti-viral signaling. Exposure to H_2_O_2_ dose-dependently reduced poly(I:C)-induced IFNβ expression (Figure 3A) and tended to reduce IFNλ expression as well (Figure 3B). To determine if reduced expression of IFNβ was due to cytotoxic effects of H_2_O_2_, levels of lactate dehydrogenase (LDH) were measured in cell-free supernatants. There was no significant increase in LDH levels by H_2_O_2_ treatment (Supplementary Figure 1). As poly(I:C) is a known TLR3 agonist we next investigated if exposure to H_2_O_2_ would affect TLR3 expression. Gene expression of TLR3 was not induced by poly(I:C) stimulation. H_2_O_2_ had a marginal effect on TLR3 gene expression in both unstimulated and poly(I:C)-stimulated bronchial epithelial cells (Figure 3C). On protein level poly(I:C) activated TLR3, noticeable through a weight shift toward 130 kDa. However, pre-treatment with H_2_O_2_ reduced the poly(I:C)-induced heavy-glycosylated TLR3. Notably, all poly(I:C)-induced fragmentation of TLR3 was reduced by H_2_O_2_ pre-treatment (Figure 3D).

### Poly(I:C)-Induced Expression of RIG-I Like Helicases Is Decreased Upon H_2_O_2_ Pre-treatment in Asthma Bronchial Epithelial Cells

**Figure 4:**
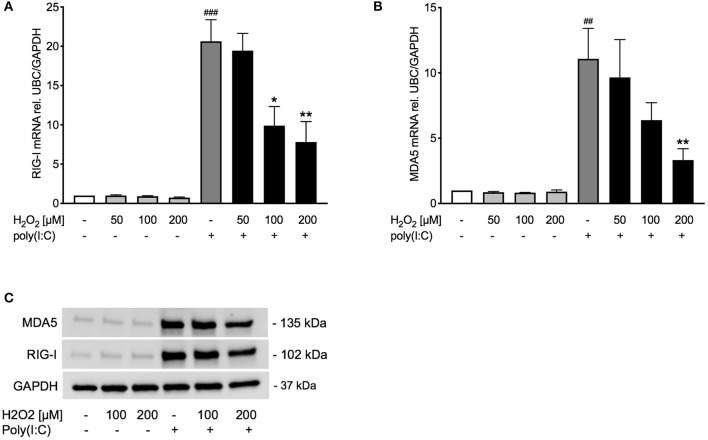
Poly(I:C)-induced expression of RIG-I like helicases is reduced upon pre-treatment with H_2_O_2_. HBECs from asthma patients were pre-treated with H_2_O_2_ for 30 min followed by stimulation with poly(I:C). Cells were harvested for gene and protein expression analysis after 3 and 24 h, respectively. Gene expression levels of RIG-I **(A)** and MDA5 **(B)** were measured by real-time PCR and data is presented as mean ± standard error of the mean (SEM) fold change of unstimulated control relative to UBC/GAPDH expression. Comparison of different groups was performed by Kruskal-Wallis with Wilcoxon post-testing. ^##^*p* < 0.01, ^###^*p* < 0.001 vs. un-stimulated (CTRL); *p < 0.05, **p < 0.01 vs. poly(I:C). Data was obtained from 7 donors. A representative western blot image (data from 2 donors) of RIG-I and MDA5 **(C)**.

Observing an effect of H_2_O_2_ pre-treatment on TLR3 gene and protein expression, we sought to study if other pattern recognition receptors involved in inducing IFNβ, such as RIG-I like helicases (4), are likewise affected. Pre-treatment with H_2_O_2_ dose-dependently decreased poly(I:C)-induced expression of the RIG-I like helicases RIG-I and MDA5 on gene (Figures 4A,B) and protein level (Figure 4C). There was a significant correlation between RIG-I mRNA and IFNβ mRNA expression and between MDA5 mRNA and IFNβ mRNA expression (Supplementary Figure 2).

### Pre-treatment With H_2_O_2_ Modestly Reduces Anti-viral Responses to Poly(I:C) in Bronchial Epithelial Cells of COPD Donors

**Figure 5:**
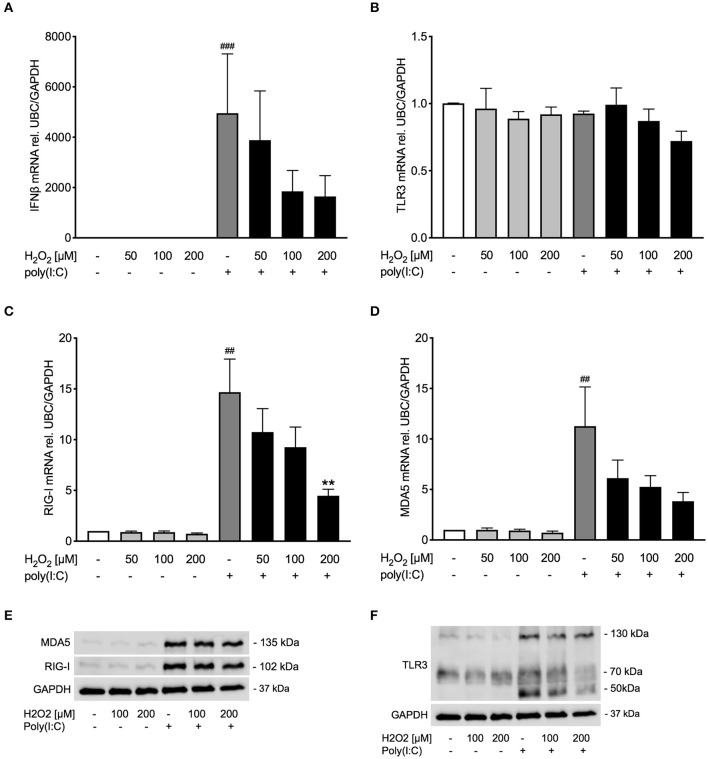
Pre-treatment with H_2_O_2_ tends to impair poly(I:C)-induced anti-viral responses in COPD bronchial epithelium. HBECs from COPD patients were pre-treated with H_2_O_2_ for 30 min followed by stimulation with poly(I:C). Cells were harvested for gene and protein expression analysis after 3 and 24 h, respectively. Gene expression levels of IFNβ **(A)**, TLR3 **(B)**, RIG-I, **(C)** and MDA5 **(D)** were measured by real-time PCR and data is presented as mean ± standard error of the mean (SEM) fold change of unstimulated control relative to UBC/GAPDH expression. Comparison of different groups was performed by Kruskal-Wallis with Wilcoxon post-testing. ^##^*p* < 0.01, ^###^*p* < 0.001 vs. un-stimulated (CTRL); **p < 0.01 vs. poly(I:C). Data was obtained from 6 donors. A representative western blot image (data from 2 donors) of RIG-I and MDA5 **(E)** and TLR3 **(F)** protein is shown.

Occurrence of oxidative stress is considered a hallmark of COPD (32), and viral infection is a major factor in exacerbations of COPD and asthma (1, 33). Hence, it was thought of interest to examine whether oxidative stress reduced poly(I:C)-induced anti-viral responses also in bronchial epithelial cells from COPD patients. Similar to bronchial epithelial cells from asthmatics, pre-treatment with H_2_O_2_ showed a trend toward dose-dependent reduction of poly(I:C)-induced expression of IFNβ in COPD bronchial epithelium (Figure 5A). The reduced IFNβ expression was associated with a decrease of RIG-I like helicases both on gene level and to some extent on protein level (Figures 5C–E). TLR3 gene expression was not affected by H_2_O_2_ pre-treatment (Figure 5B) but poly(I:C)-induced TLR3 protein expression was inhibited by H_2_O_2_ (Figure 5F). The more modest effects of oxidative stress on poly(I:C)-induced anti-viral responses in COPD patients was mirrored in their expression of SOD1 and SOD2 which were little affected by H_2_O_2_ pre-treatment (Supplementary Figure 3). Further, there was no difference in epithelial IFNβ gene expression at baseline or after poly(I:C) stimulation between the asthma and COPD donors (Supplementary Figure 4).

### Knockdown of TLR3 Abrogates the Impairment of IFNβ Expression by H_2_O_2_

**Figure 6:**
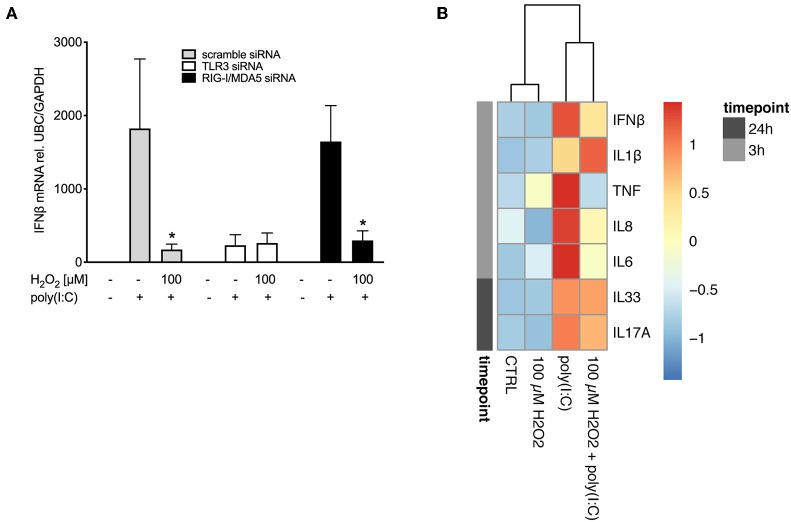
H_2_O_2_ impairs responsiveness to TLR3 and reduces cytokine responses to poly(I:C). HBECs were pre-treated with H_2_O_2_ for 30 min followed by stimulation with poly(I:C). **(A)** Cells were exposed to siRNA directed against TLR3 or RIG-I like helicases or to non-specific siRNA (scramble) before start of the experiment. Cells were harvested 3 h post poly(I:C) stimulation for gene expression analysis. Gene expression levels of IFNβ were measured by real-time PCR and data is presented as mean ± standard error of the mean (SEM) fold change of unstimulated control relative to UBC/GAPDH expression. Comparison of different groups was performed by Kruskal-Wallis with Wilcoxon post-testing. *p < 0.05 vs. poly(I:C). Data was obtained from 8 asthma/COPD donors. **(B)** Cell-free supernatants were obtained 3 and 24 h post poly(I:C) stimulation. Cytokine levels of IFNβ, IL-1β, TNF, IL-8, IL-6, IL-33, and IL-17A were measured with multiplex ELISA and expressed as protein concentration in pg/ml. Data was obtained from 5 asthma donors.

As poly(I:C)-induced expression of pattern recognition receptors was affected by pre-treatment with H_2_O_2_ we sought to investigate if the effect of H_2_O_2_ on IFNβ expression might be due to its effects on pattern recognition receptor expression. Knockdown of pattern recognition receptors was confirmed (Supplementary Figure 5). Double knockdown of the RIG-I like helicases RIG-I and MDA5 did not affect H_2_O_2_-induced impairment of poly(I:C)-induced IFNβ expression and resulted in a similar expression pattern as knockdown with scramble siRNA (Figure 6A). In contrast, when TLR3 was knocked down pre-treatment with H_2_O_2_ did not further reduce poly(I:C)-induced IFNβ expression (Figure 6A), suggesting involvement of TLR3 in the effects of H_2_O_2_ on the anti-viral responses.

### Pre-treatment With H_2_O_2_ Reduces Expression of Poly(I:C)-Induced Cytokines in Asthma Bronchial Epithelial Cells

We next investigated if oxidative stress affected expression of other cytokines relevant to asthma. We observed that pre-treatment with H_2_O_2_ decreased poly(I:C)-induced IFNβ protein levels (Figure 6B), confirming our findings on gene level (Figure 3A). Further, oxidative stress reduced protein levels of poly(I:C)-induced IL-6, IL-8 and TNF, while increasing poly(I:C)-induced expression of IL-1β. There was no effect of oxidative stress on IL-33 and IL-17A protein levels (Figure 6B) in asthma bronchial epithelial cells.

## Discussion

Levels of reactive oxygen species are enhanced in asthmatic airways as a result of inflammation (11) and are inversely correlated with lung function (34). Activity of the antioxidant enzymes is also reduced in asthmatic bronchoalveolar lavage fluid and airway epithelial cells (35, 36). The disequilibrium in oxidation state of the lungs is associated with asthma severity (37). During exacerbations, frequently involving viral infections, levels of reactive oxygen species are further increased (27, 28). In COPD oxidative stress is considered a major disease mechanism (17) and oxidative stress markers in sputum are further increased at exacerbation (38). Our results of impaired viral stimulus-induced expression of antioxidant enzymes in diseased epithelium is consistent with these clinical observations. Further, we demonstrate here that oxidative stress in epithelial cells from diseased donors reduces anti-viral responses possibly by reducing responsiveness to TLR3.

It has been previously shown that experimental rhinovirus infection induces oxidative stress in mild asthmatics, particularly in those with high eosinophil counts. Depletion of eosinophils resulted in reduced oxidative stress after rhinovirus infection in this group, demonstrating the role of oxidative stress at exacerbations (39). In this study, both live rhinovirus infection and stimulation with the synthetic viral replication intermediate poly(I:C) induces antioxidant expression in bronchial epithelial cells obtained from diseased individuals. Considering the need for a model that mimics both the initial impact of viral infection, which produces an oxidative burden, but also the ensuing infectious state we pre-treated bronchial epithelial cells from asthmatics and COPD patients with H_2_O_2_ to produce a low level oxidative stress followed by stimulation with poly(I:C) as an infection-like burden. These interaction experiments did not stimulate cell death and release of cellular material as suggested by LDH measurements. The H_2_O_2_-poly(I:C) co-stimulation dose-dependently reduced gene and protein levels of SOD2 and partially SOD1, thus mimicking clinical findings of antioxidant deficiency at asthma exacerbation (40). This data supports the potential relevance of our *in vitro* model.

Deficiency of anti-viral interferons has been observed in many cohorts of asthmatics (6–8). However, mechanisms causing interferon deficiency are largely unknown. We have previously shown that house dust mite impaired anti-viral responses at experimental asthma exacerbations *in vitro* and *in vivo* (41). Proteolytic allergens, such as house dust mite, are a known source of reactive oxygen species (16, 42), suggesting that oxidative stress could have been involved in the reduced anti-viral responses that we observed (41). Here we show that asthmatic bronchial epithelial cells pre-treated with H_2_O_2_ have reduced IFNβ expression upon poly(I:C) stimulation. Reduction of poly(I:C)-induced IFNβ expression by H_2_O_2_ was associated with decreased expression of RIG-I like helicases in our model. TLR3, which is constitutively expressed (43), was only marginal effected by H_2_O_2_ on gene level. On protein level we observed a fragmentation of TLR3 into its C-terminal fragment (present already at baseline) as well as its N-terminal fragment [stimulated by poly(I:C)] (44). Further, poly(I:C) induced expression of heavy glycosylated full-length TLR3, its signaling-active form (45). Co-stimulation of H_2_O_2_ and poly(I:C) reduced expression of all fragments.

Further, oxidative stress induced protein levels of IL-1β upon poly(I:C) stimulation, a cytokine which is involved in driving inflammation and a predictor for future asthma exacerbations (46). In contrast poly(I:C)-induced protein expression of IL-8 and TNF was reduced by pre-treatment with H_2_O_2_, thus predisposing toward a reduced immune response against infectious agents (47).

We further investigated the effect of H_2_O_2_ on anti-viral responses in bronchial epithelial cells obtained from COPD patients. Similarly to asthma the COPD epithelium responded with reduced expression of IFNβ and pattern recognition receptors to oxidative stress, however, the effects were more modest compared to the asthmatic epithelium included in this study. Further, the oxidative stress produced in our model did not affect expression of the antioxidants SOD1 and SOD2 in the COPD epithelium. As oxidative stress is considered a major player in the pathogenesis of COPD (32) it is possible that a high oxidant burden in COPD (48) may make epithelial cells less responsive to further oxidative stress.

We demonstrate that pre-treatment with H_2_O_2_ reduced poly(I:C)-induced pattern recognition receptor expression, which is upstream of IFNβ. Hence, we hypothesized that H_2_O_2_ might directly affect PRR expression. Employing knockdown studies using siRNA we demonstrated that knockdown of TLR3 completely abrogated poly(I:C)-induced IFNβ expression, while knockdown of RIG-I/MDA5 had no effect on poly(I:C)-induced IFNβ, thus demonstrating the importance of TLR3 for IFNβ signaling. Further, absence of RIG-I/MDA5 did not result in alteration of H_2_O_2_-induced impairment of poly(I:C)-induced IFNβ expression. While these findings need further exploration, they hint at a possible involvement of TLR3 in H_2_O_2_-induced impairment of poly(I:C)-induced IFNβ expression. The present reduction of poly(I:C)-induced RIG-I like helicase expression by H_2_O_2_ might thus be due to a IFNβ-mediated feedback mechanism (49).

To our knowledge this is the first study investigating innate immune responses in an *in vitro* model of H_2_O_2_-poly(I:C) co-stimulation using primary bronchial epithelial cells. However, as the number of included patients was low further studies are needed to elucidate if the presented findings can be replicated in a larger patient cohort. Further, thorough quantification of the effects of H_2_O_2_-poly(I:C) co-stimulation on protein expression of anti-viral responses is warranted.

**Figure 7:**
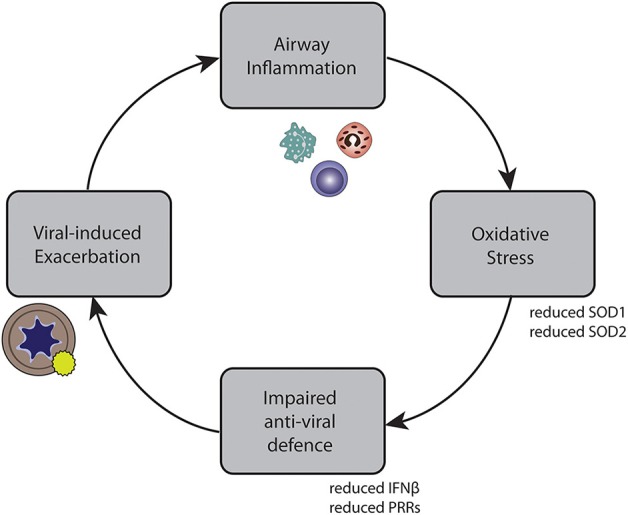
Self-perpetuating circle of inflammation, impaired anti-viral defense, exacerbations, and inflammation in asthma and COPD.

In conclusion, we developed a method by which we could show that oxidative stress attenuates anti-viral responses in primary bronchial epithelial cells of asthmatic and COPD patients. These findings could contribute to a better understanding of reduced anti-viral signaling observed at viral-induced exacerbations. Together, our findings suggest a possible self-perpetuating vicious cycle underlying recurrent exacerbations, leading to an impaired anti-viral response, which in turn leads to viral-induced exacerbations, causing more airway inflammation (Figure 7). This might provide part of the explanation for the increased risk of recurrent exacerbations and exacerbation relapses observed in patients with eosinophilic airway inflammation (50). They may further suggest that in order to stabilize asthma patients with recurrent exacerbations, treatment approaches that reduce oxidative stress may be relevant.

## Data Availability Statement

The datasets generated for this study are available on request to the corresponding author.

## Ethics Statement

The studies involving human participants were reviewed and approved by the regional ethical review board at Lund University and the review board in Copenhagen. The patients/participants provided their written informed consent to participate in this study.

## Author Contributions

MM and LU contributed to the conception and design of the work. MM, SR, JC, HA, BO, and LU contributed to the acquisition, analysis, and interpretation of the work. ET, LB, AS, and CP provided clinical samples. MM drafted the manuscript. MM, SR, JC, BO, AS, CP, ET, LB, HA, and LU revised the manuscript. All authors have read and approved the submission of the manuscript.

### Conflict of Interest

The authors declare that the research was conducted in the absence of any commercial or financial relationships that could be construed as a potential conflict of interest.
