# Supplementary material for: Oxidative Stress Attenuates TLR3 Responsiveness and Impairs Anti-viral Mechanisms in Bronchial Epithelial Cells From COPD and Asthma Patients
Source: Front Immunol. 2019 Nov 29;10:2765. doi: 10.3389/fimmu.2019.02765 (PMC6895140; doi:10.3389/fimmu.2019.02765)
Supplement: Supplementary file 1 [file Data_Sheet_1.docx]

Supplementary Material

#

**Supplementary Figure 1. Pre-treatment with H_2_O_2_ does not induced LDH release.** HBECs from COPD and asthma patients were pre-treated with H_2_O_2_ for 30 min followed by stimulation with poly(I:C). Lactate dehydrogenase levels were measured in cell-free supernatants 3h (A) and 24h (B) post poly(I:C) stimulation and related to total protein content in the supernantant. Data is presented as mean ± standard error of the mean (SEM). Comparison of different groups was performed by Kruskal-Wallis with Wilcoxon post-testing. Data was obtained from 6-8 donors.

Supplementary Figure 2. poly(I:C)-induced RIG-I and MDA5 gene expression after pre-treatment with H_2_O_2_ correlates with IFNβ gene expression. HBECs from asthma patients were pre-treated with H_2_O_2_ for 30 min followed by stimulation with poly(I:C). Correlations were analysed by Spearman. For correlation with a P-value below 0.05, and thus statistically significant, linear regression was employed.

Supplementary Figure 3. Pre-treatment with H_2_O_2_ does not alter poly(I:C)-induced SOD1 and SOD2 expression in COPD bronchial epithelium. HBECs from COPD patients were pre-treated with H_2_O_2_ for 30 min followed by stimulation with poly(I:C). Cells were harvested for gene and protein expression analysis after 3h and 24h, respectively. Gene expression levels of SOD2 (A) and SOD1 (B) were measured by real-time PCR and data is presented as mean ± standard error of the mean (SEM) fold change of unstimulated control relative to UBC/GAPDH expression. Comparison of different groups was performed by Kruskal-Wallis with Wilcoxon post-testing. #p < 0.05, ##p < 0.01 vs CTRL. Data was obtained from 6 donors. A representative western blot image (data from 2 donors) of SOD1 and SOD2 (C) protein is shown.

Supplementary Figure 4. No difference in IFNβ expression at baseline or after poly(I:C) stimulation between COPD and asthma patients. HBECs from asthma and COPD patients were stimulated with poly(I:C). Cells were harvested for gene expression analysis after 3h. Gene expression levels of were measured by real-time PCR and data is presented as mean ± standard error of the mean (SEM). Gene expression is expressed as Ct of IFNβ related to Ct of UBC/GAPDH (HKG). Comparison of different groups was performed by Kruskal-Wallis. Data was obtained from 6-7 donors.

**Supplementary Figure 5. Confirmation of knockdown of pattern recognition recptors by siRNA.** Diseased HBECs were exposed to siRNA directed against TLR3 or RIG-I like helicases or to non-specific siRNA (scramble). Cells were then pre-treated with H_2_O_2_ for 30 min followed by stimulation with poly(I:C). A representative western blot image of TLR3, RIG-I and MDA5 protein is shown.

**
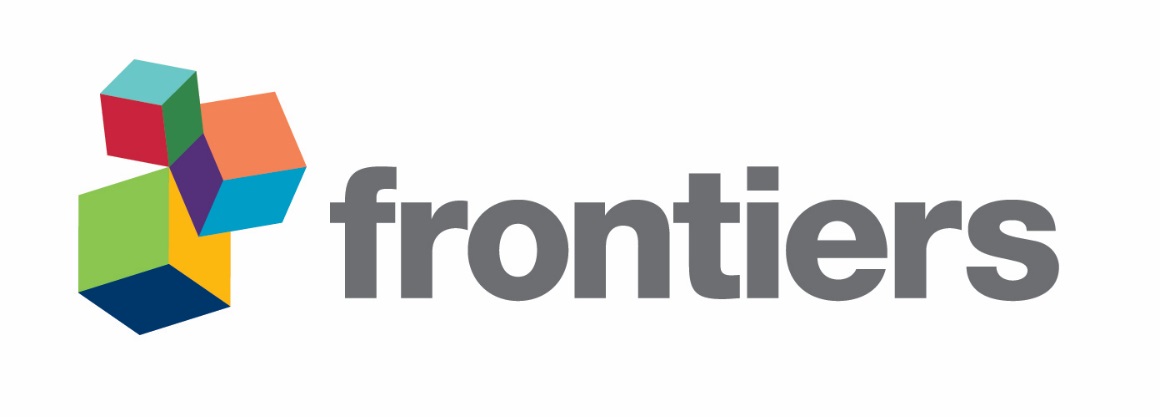
**
